# Supplementary figures and images for: Comparative Analysis of Three Different Types of Fermented Tea by Submerged Fermentation with Eurotium cristatum
Source: Foods. 2025 Sep 18;14(18):3241. doi: 10.3390/foods14183241 (PMC12469720; doi:10.3390/foods14183241)

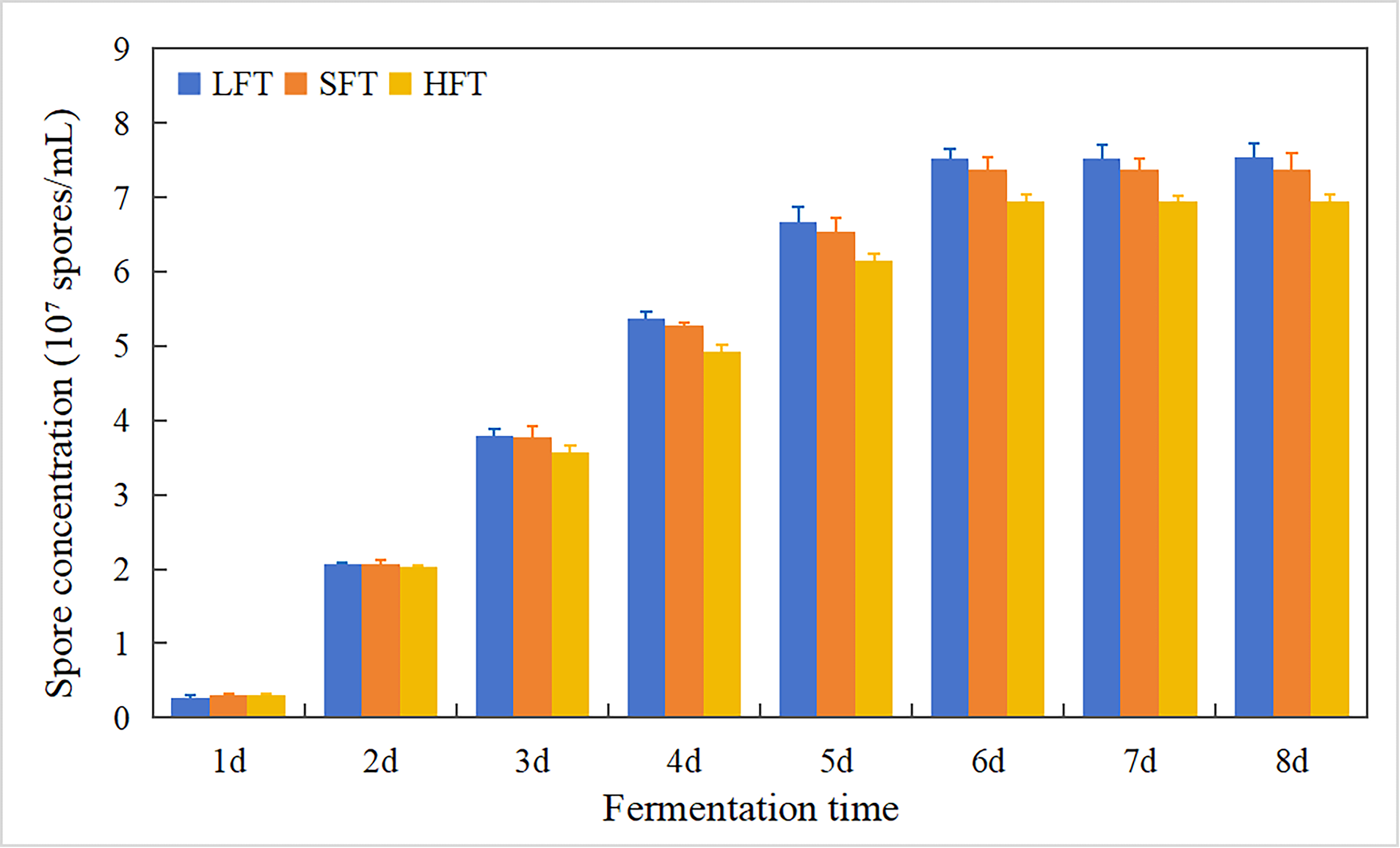

Supplement: Supplementary file 1 [file foods-14-03241-s001.zip › Supplementary Fig.1.jpg]

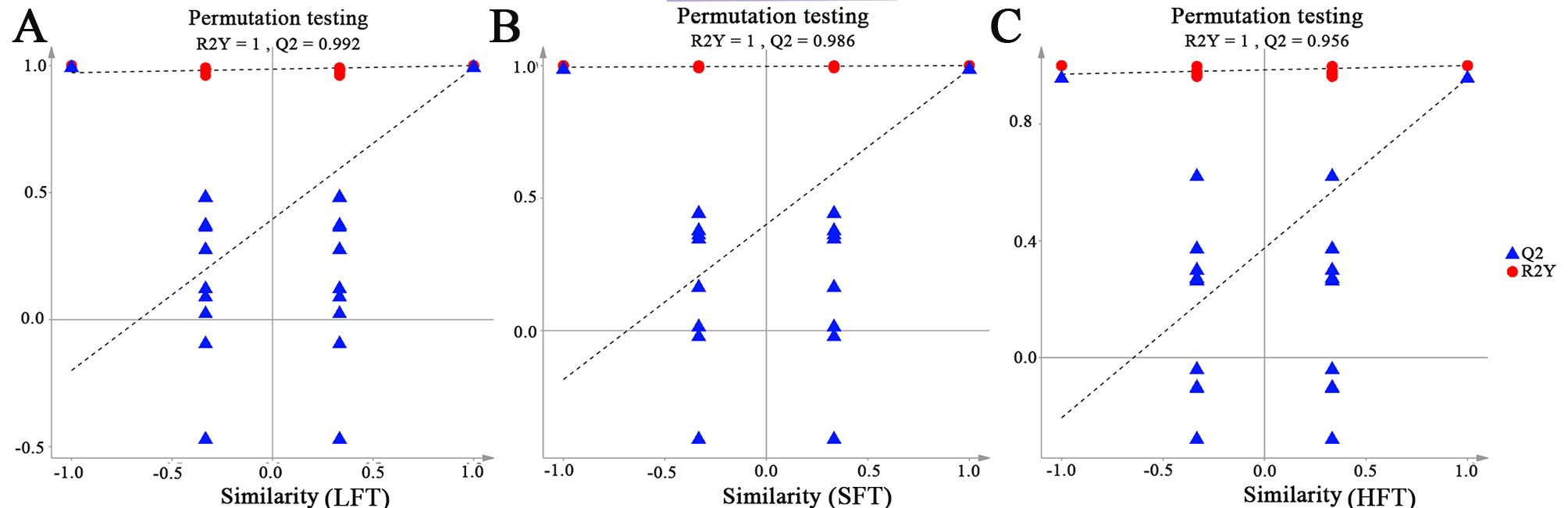

Supplement: Supplementary file 1 [file foods-14-03241-s001.zip › Supplementary Fig.2.jpg]

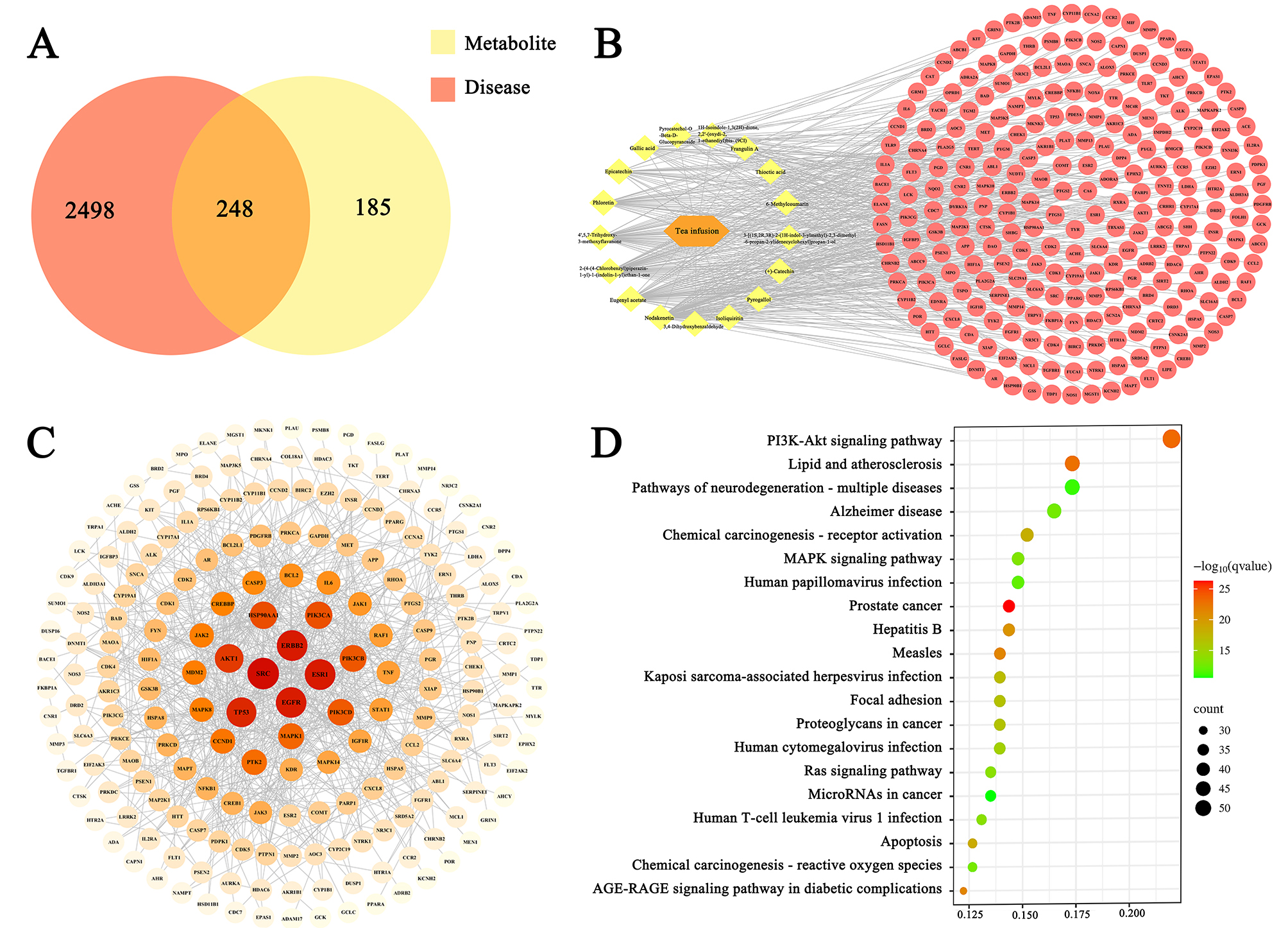

Supplement: Supplementary file 1 [file foods-14-03241-s001.zip › Supplementary Fig.3.jpg]
